# Duffy Antigen Receptor for Chemokines Regulates Post-Fracture Inflammation

**DOI:** 10.1371/journal.pone.0077362

**Published:** 2013-10-17

**Authors:** Charles H. Rundle, Subburaman Mohan, Bouchra Edderkaoui

**Affiliations:** 1 Musculoskeletal Disease Center, Research Service, Jerry L Pettis Memorial Veterans Administration Medical Center, Loma Linda, California, United States of America; 2 Department of Medicine, Loma Linda University, Loma Linda, California, United States of America; 3 Department of Biochemistry, Loma Linda University, Loma Linda, California, United States of America; 4 Department of Physiology, Loma Linda University, Loma Linda, California, United States of America; University of Texas Health Science Center San Antonio Texas, United States of America

## Abstract

There is now considerable experimental data to suggest that inflammatory cells collaborate in the healing of skeletal fractures. In terms of mechanisms that contribute to the recruitment of inflammatory cells to the fracture site, chemokines and their receptors have received considerable attention. Our previous findings have shown that Duffy antigen receptor for chemokines (*Darc*), the non-classical chemokine receptor that does not signal, but rather acts as a scavenger of chemokines that regulate cell migration, is a negative regulator of peak bone density in mice. Furthermore, because *Darc* is expressed by inflammatory and endothelial cells, we hypothesized that disruption of *Darc* action will affect post-fracture inflammation and consequently will affect fracture healing. To test this hypothesis, we evaluated fracture healing in mice with targeted disruption of *Darc* and corresponding wild type (WT) control mice. We found that fracture callus cartilage formation was significantly greater (33%) at 7 days post-surgery in *Darc*-KO compared to WT mice. The increased cartilage was associated with greater Collagen (*Col*) II expression at 3 days post-fracture and *Col*-X at 7 days post-fracture compared to WT mice, suggesting that *Darc* deficiency led to early fracture cartilage formation and differentiation. We then compared the expression of cytokine and chemokine genes known to be induced during inflammation. Interleukin (*Il*)-1β, *Il-6*, and monocyte chemotactic protein 1 were all down regulated in the fractures derived from *Darc*-KO mice at one day post-fracture, consistent with an altered inflammatory response. Furthermore, the number of macrophages was significantly reduced around the fractures in *Darc*-KO compared to WT mice. Based on these data, we concluded that *Darc* plays a role in modulating the early inflammatory response to bone fracture and subsequent cartilage formation. However, the early cartilage formation was not translated with an early bone formation at the fracture site in *Darc*-KO compared to WT mice.

## Introduction

Fracture healing is a complex process that involves the interaction of soluble mediators, extracellular matrix components, resident cells and infiltrating leukocyte subtypes, which participate differentially in the classically defined three phases of fracture healing: inflammation, bone formation and bone remodeling [1], [2]. In the inflammatory phase, a fibrin mesh develops and acts as a scaffold for the infiltrating neutrophils and macrophages to clear the tissue debris. A cytokine/chemokine cascade initiates the proliferation of fibrous cells in the fracture gap that differentiate to cartilage and cancellous bone, and thereafter remodel into lamellar bone through the coordinated action of bone forming osteoblast cells and bone resorbing osteoclast cells [3], [4], [5], [6]. The inflammatory phase that initiates this process is critical for successful bone repair, and its elucidation will identify new approaches to enhance bone repair in normal and impaired conditions.

The role of some of the chemokine receptors has been investigated in fracture healing [7]. However, the role of the duffy antigen receptor for chemokines (*Darc*), which is known to play an important role in chemokine-regulated leukocyte/neutrophil trafficking during inflammation has not been characterized in fracture healing. We have previously identified *Darc* as a negative regulator of bone mineral density [8]. *Darc* is known to bind chemokines that regulate cell trafficking [9]. It is highly expressed in erythrocytes as well as vascular endothelial cells [10], [11], the cell types that play key role in wound healing process [12], [13], [14]. Based on the established role of inflammation in fracture healing, and the predicted role of *Darc* in regulating function of inflammatory chemokines, we proposed that *Darc* expression plays an important role in post-fracture inflammation and fracture healing. To test this hypothesis, we have used *Darc*-KO mice and wild type mice to compare femur fracture healing and the post-fracture expression levels of inflammatory genes in the two lines of mice.

## Materials and Methods

### Femur Fracture Model

All procedures were performed with the approval of the institutional Animal Care and Use Committee (IACUC) in accordance with the Animal Welfare Act at the Jerry L. Pettis Memorial VA Medical Center, Loma Linda, CA, USA. Dr. A. Chaudhuri (New York Blood Center, NY, USA) provided the *Darc*-knockout (*Darc*-KO) mice, as well as the control wild type (WT) mice. Mice were generated as previously described by Luo et al. [15], and were bred and maintained as previously described [8].

Ten to twelve week old mice were placed under isoflurane anesthesia and femoral fractures were produced by the three-point bending technique, as previously described [16], [17]. Briefly, the femur was internally stabilized with a 0.5 mm diameter stainless steel pin surgically implanted prior to fracture, and a 4-0 PDSII suture was used to close the wound. Buprenorphine was administered subcutaneously after fracture for pain relief. Animals were allowed unrestricted movement during post-fracture healing. Tissues were harvested for analysis at different intervals of post-fracture healing.

### Histomorphometry

Histomorphometry analyses were performed at 7, 11 and 21 days post-fracture. Bones were fixed in 10% formalin, demineralized in EDTA, paraffin-embedded and each sample was sectioned longitudinally at 5–6 µm thickness as described by Bancroft [18]. Sections were stained with Safranin-Orange and Fast Green to assess cartilage area and total callus area. Data are reported as the mean of callus area and cartilage area, and excluded the native cortical bone and the intramedullary space. Quantification was performed using Image Pro software 6.3 (Media Cybernetics, Silver Spring, MD, USA).

### X-ray analysis

Fracture repair was analyzed by X-ray examination using a micro-computed tomography scanner (μ-CT; *Viva*Ct 40 scanner, SCANCO Medical AG, Brüttisellen, Switzerland). Analyses were performed on fractured and unfractured bones at 21 days post-fracture, when healing of the fractured bone in the wild-type mouse normally approaches bony union of the fracture callus. Cortical bone and the surrounding mineralized tissue of the fracture callus were manually contoured. Total tissue volume was defined as the circumference of the bony callus. To normalize the length of bone examined, the analysis was performed on a standard length around the fracture, encompassing 591±5 slices of the scanned image and centered at the fracture site. We used two thresholds to differentiate between lower density callus cancellous bone (220–570 mg HA/cm^3^) and native cortical bone (570–1000 mg HA/cm^3^). To normalize for trabecular bone contributions to the fracture callus measurements, the unfractured bone trabecular values were subtracted from the fracture callus data.

### Gene Expression Analysis

Animals were sacrificed at 1, 3, 7 and 15 days post-surgery. Total RNA was isolated from fracture tissues as follows: Approximately 2 mm from each side of the fracture was quickly harvested and the fracture callus including bone marrow was stored in liquid nitrogen. The callus was then pulverized with Trizol under cryogenic conditions and following the protocol provided by Invitrogen. We have found this approach to be very successful in maintaining RNA integrity during purification. Reverse transcription was performed with MMLV Reverse Transcriptase (Promega, San Luis Obispo, CA, USA). Real-time PCR was performed using the SYBRgreen master mix (Applied Biosystems, Foster City, CA, USA) with gene-specific primers (Integrated DNA Technologies, Coralville, IA, USA). The information about the sequence of the primers used in this study is presented in Table S1. Changes in gene expression were determined by subtracting the Ct (threshold cycle) of target gene from the Ct value of the housekeeping gene; peptidylprolyl isomerase A (*Ppia*) (ΔCt = Ct of target gene – Ct of *Ppia*) as described in Table S2. Mean ΔCt was then used to calculate the difference in cycle thresholds between the wild-type (WT) unfractured bones and WT or *Darc*-KO fractures (ΔΔCt = mean ΔCt of WT unfractured bones - mean ΔCt of fractured bones from each genotype). The fold-activation was calculated as 2^−ΔΔCt^ [19]. The genes examined were as follows: Receptor activator of nuclear factor-κB ligand (*Rankl*), osteoprotegerin (*Opg*), tumor necrosis factor (*Tnf*)-α, interleukin (*Il*)-1beta (*β*), *Il-6*, macrophage inflammatory protein-1 alpha (*Mip*-1α/*Ccl3*), monocyte chemotactic protein-1 (*Mcp*-1/*Ccl2*), as well as collagen II (*Col-*II) and collagen X (*Col*-X).

### Detection and quantification of inflammatory cells in fracture tissues

Immunohistochemistry was performed using rat anti-mouse Ly-6B.2, F4/80 (ABD Serotec, A Divison of MorphoSys, Raleigh, NC, USA), and CD45R (BD Pharmingen, San Jose, CA, USA) to identify neutrophils, macrophages, and B lymphocytes, respectively. The longitudinal sections prepared as described above were incubated with primary antibodies for 60 min at 4°C. Conjugate and substrate were used according to manufacturer instructions and following the protocol provided by BIOCARE Medical (Biocare Medical, Concord, CA, USA). The number of positive cells in the fracture tissues was determined using OsteoMeasure software (Osteometrics Inc. GA, USA). Values were expressed as number of cells per mm^2^ tissue.

### Statistical analysis

Statistical significance was evaluated using two-tailed Student's *t-test*. The difference is considered statistically significant, when *p*<0.05.

## Results

### Lack of *Darc* expression enhanced post-fracture cartilage formation

**Figure 1:**
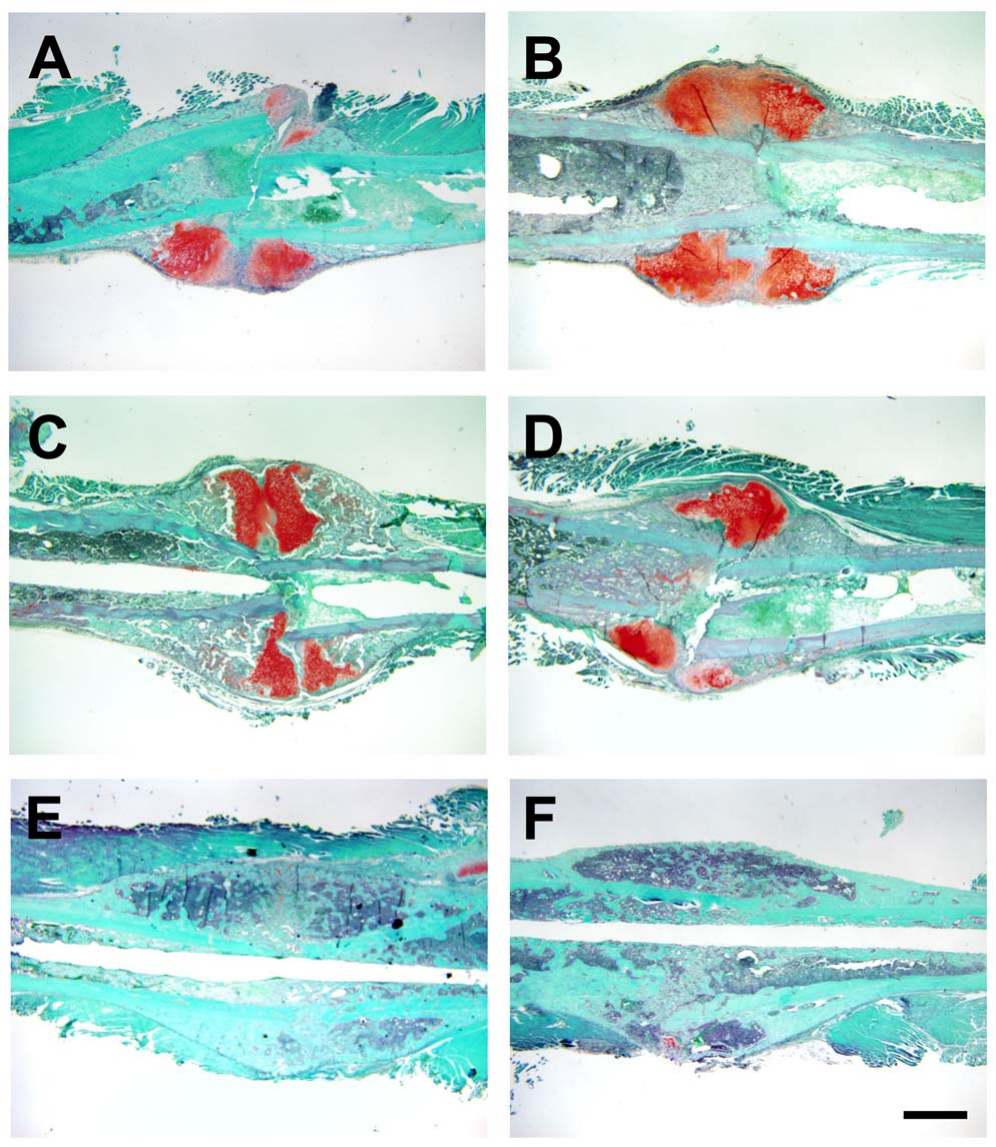
Callus and cartilage development during fracture healing in WT and *Darc*-KO mice. Cartilage was stained with Safranin-Orange in fracture calluses derived from WT (**A, C, E**) and *Darc*-KO mice (**B, D, F**) at 7 (**A, B**), 11 (**C, D**) and 21 days post-fracture (**E, F**). Scale bar = 1 mm.

**Figure 2:**
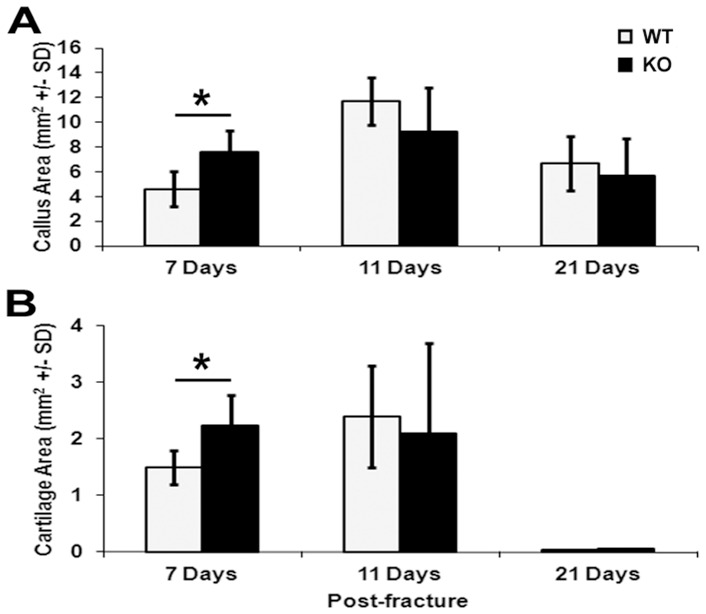
Quantification of callus and cartilage development during fracture healing. Histomorphometric quantification of cartilage area (**A**) and fracture callus areas (**B**) derived from WT and *Darc*-KO mice at 7, 11 and 21 days post-fracture. We examined 5–9 animals/time point/strain of mice. *p<0.05 WT *vs Darc*-KO mice.

**Figure 3:**
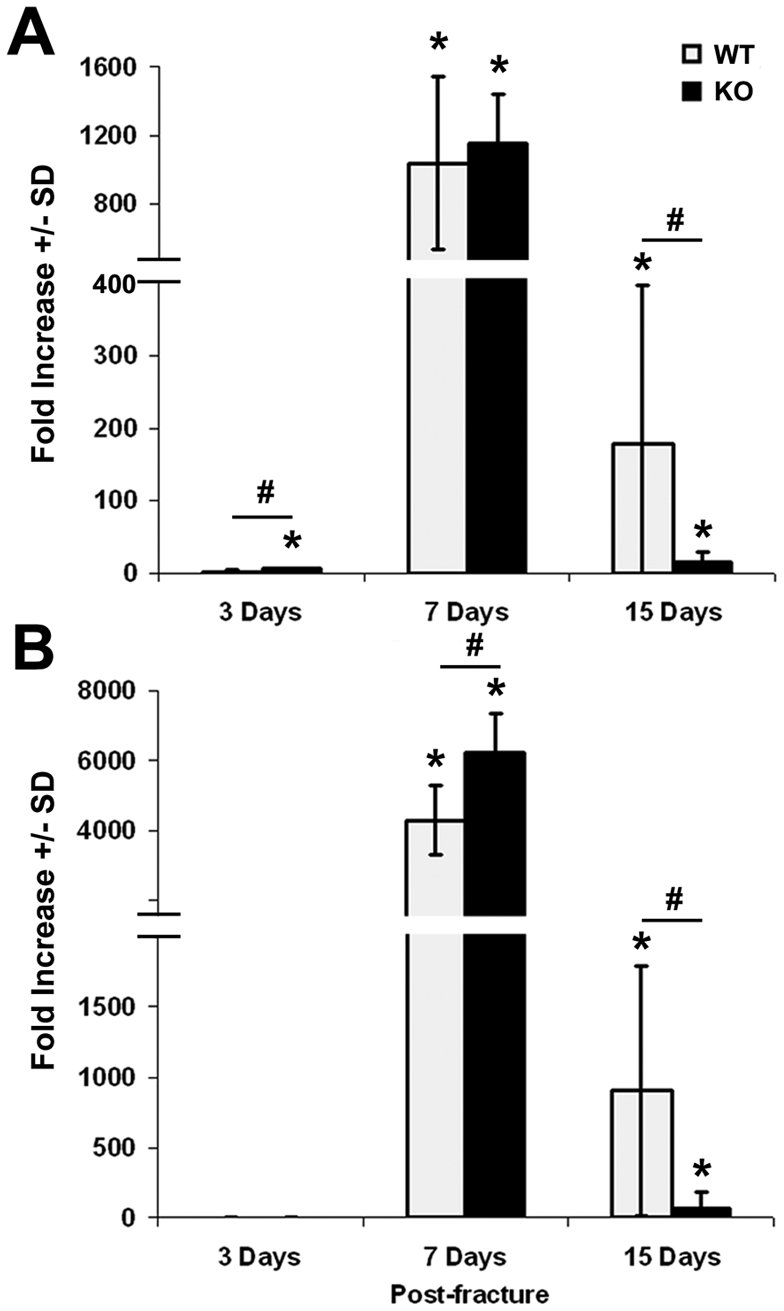
Expression of cartilage marker genes during fracture healing. Collagen II (**A**) and Collagen X (**B**) expression in fracture calluses derived from WT and *Darc*-KO mice at 3, 7 and 15 days post-fracture. Data are expressed as fold change in the expression of the gene in the fractured bones compared to unfractured bone of WT mice. We analyzed 3–4 animals/mouse strain at 3 days post-surgery and 5–8 animals/mouse strain at 7 and 15 days post-fracture.*p<0.05 *vs* WT unfractured bones, #*p*<0.05 between fractured bones of the two lines of mice.

To determine if lack of *Darc* expression affects fracture healing process, we performed histomorphometric analysis of the fracture callus cartilage in *Darc*-KO and WT mice at 7, 11 and 21 days post-fracture (**Fig. 1**). We found that fracture callus size (**Fig. 2A**) and fracture cartilage area (**Fig. 2B**) were significantly greater at 7 days post-surgery in *Darc*-KO mice compared to WT mice (45% and 33%, respectively, *p*<0.03). However, at 11 and 21 days post-fracture, the callus size was not significantly different between the two lines of mice. To evaluate fracture cartilage development, we compared the expression of *Col*-II and *Col*-X, markers of pre-hypertrophic and hypertrophic chondrocytes, respectively. We found the expression levels of both *Col*-II and *Col*-X were greater in both strains of mice at 7 days, compared to 3 and 15 days post-surgery, as would be expected in normal fracture repair (**Fig. 3**). *Darc*-KO mice exhibited greater expression of *Col*-II at 3 days post-fracture (**Fig. 3A**) and *Col*-X at 7 days post-fracture (**Fig. 3B**) compared to WT mice. At 15 days post-surgery, the expression of both genes was significantly down-regulated in fracture calluses of both lines of mice compared to 7 days post-fractures, as is expected during the late stages of fracture cartilage development.

### *Darc* deficiency did not improve fracture healing - Micro-CT data at 21 days post-fracture

**Figure 4:**
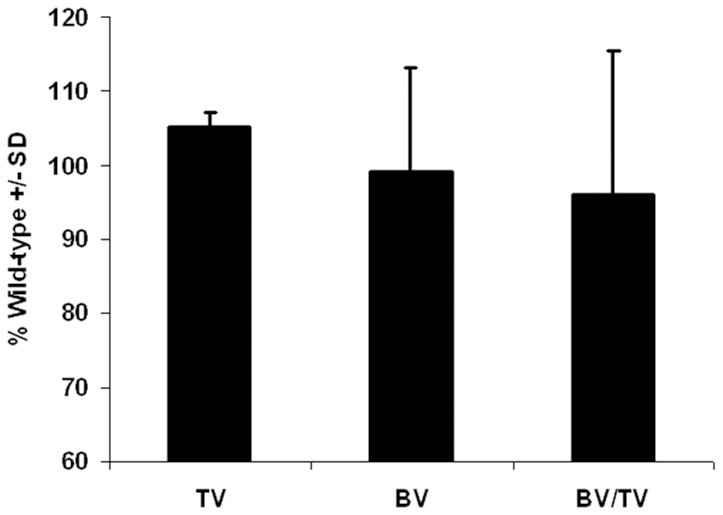
Micro-CT analysis of the fracture calluses. Data are presented as the percentage of WT. Five fractures were examined from each strain of mice. BV, bone volume; TV, total volume; BV/TV, bone volume fraction.

**Figure 5:**
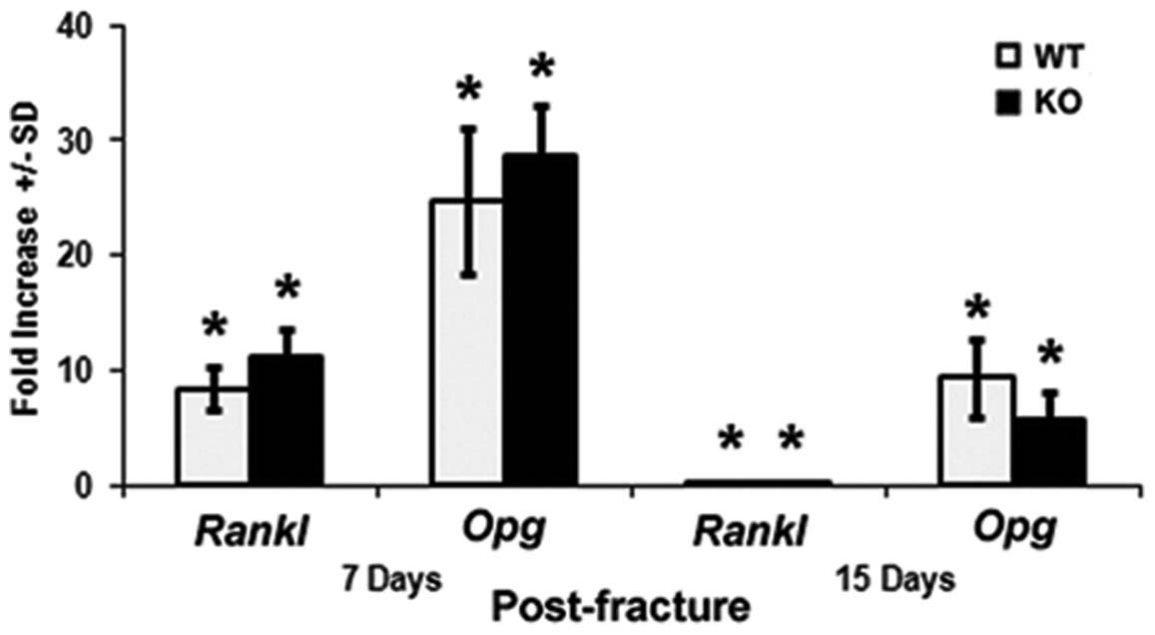
Expression of *Rankl* and *Opg* genes at 7 and 15 days post-fracture. Data are expressed as fold-change in the expression of the gene in the fractured bones compared to unfractured bones of WT mice. We analyzed 6–8 animals/mouse strain. *p<0.05 *vs* WT unfractured bones.

To determine if the early cartilage formation in *Darc*-KO mice changed callus bone formation during fracture healing, we analyzed the mineralized tissues of the hard callus by micro-CT at 21 days post-fracture. Surprisingly, no significant difference in total volume or bone volume of the fracture calluses was observed between *Darc*-KO mice and WT mice at 21 days post-fracture (**Fig. 4**). To determine if osteoclastogenesis was affected by lack of *Darc* expression; we compared the mRNA expression of *Rankl* and *Opg*, the decoy receptor at the fracture calluses between the two lines of mice (**Fig. 5**). Both genes were up regulated at 7 days post-fracture when compared to WT unfractured bones, but no significant difference was observed between the two lines of mice. At 15 days post-fracture, mRNA expression of both genes was down-regulated in fracture calluses from both lines of mice compared to 7 days post-fracture, but no difference was observed between the two lines of mice at this time point.

### *Darc* expression regulates post-fracture inflammation

**Figure 6:**
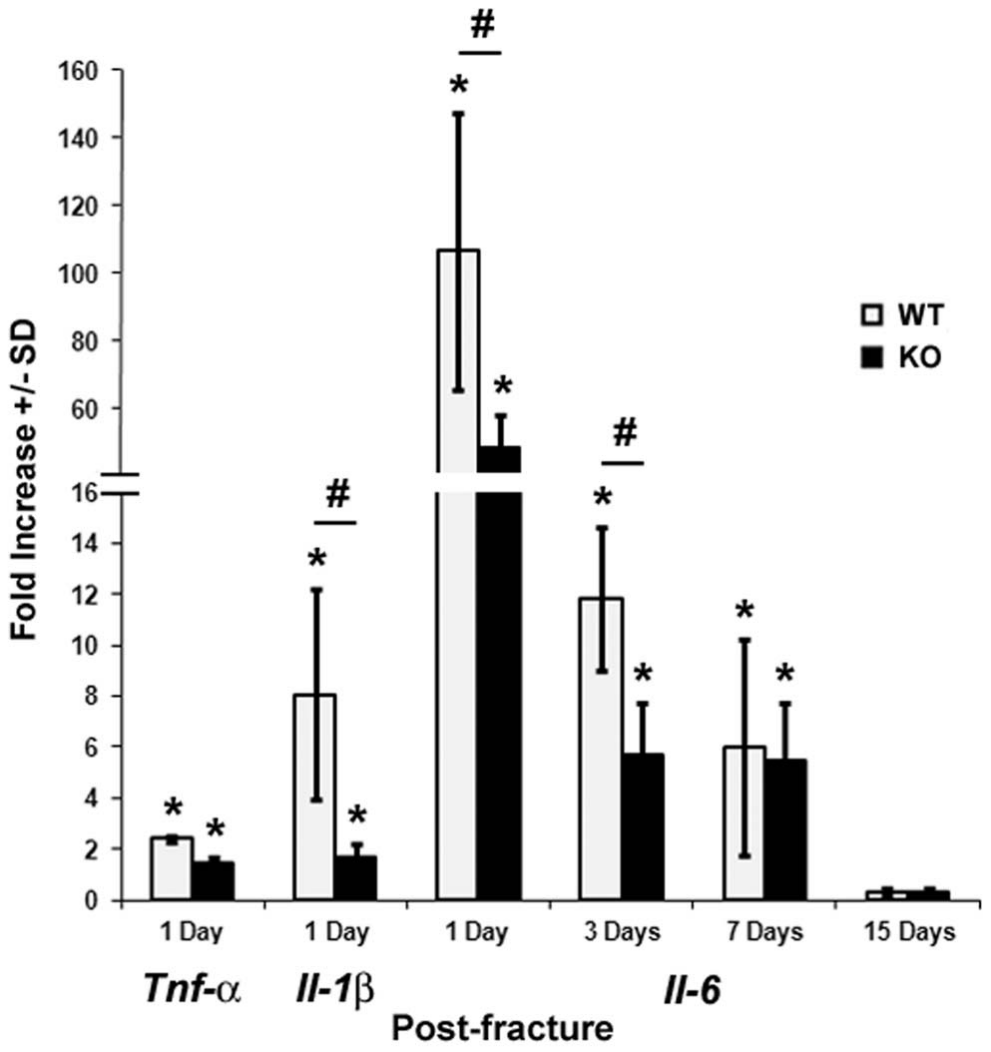
Expression of cytokine genes at four time points post-fracture. Data are expressed as fold-change in the expression of the gene in the fractured bones compared to unfractured bones of WT mice. We analyzed 3–4 animals/mouse strain at 1 and 3 days and 5–8 mice at 7 and 15 days post-fracture.*p<0.05 *vs* WT unfractured bones, #*p*<0.05 between fractured bones of the two lines of mice.

The pro-inflammatory cytokines; TNF-α, IL-1β and IL-6 have been shown not only to coordinate the hematopoietic and immune systems, but also to contribute to bone repair by regulating osteoclastogenesis and the early recruitment and differentiation of osteoblastic lineage cells [20], [21], [22], [23], [24], [25], [26], [27], [28], [29]. Therefore, we have analyzed the effect of targeted disruption of *Darc* on the expression of these three inflammatory cytokines in bone fracture. As expected, the mRNA level of the three cytokines was enhanced after one day of bone fracture in both lines of mice (**Fig. 6**), but the magnitude of increase in the expression of IL-1β and IL-6 was reduced by 52–54% in the fractures derived from *Darc*-KO mice compared to the fractures derived from WT mice at one day post-fracture. The expression levels of the three cytokines in unfractured bones were not different between the two lines of mice (data not shown).

**Figure 7:**
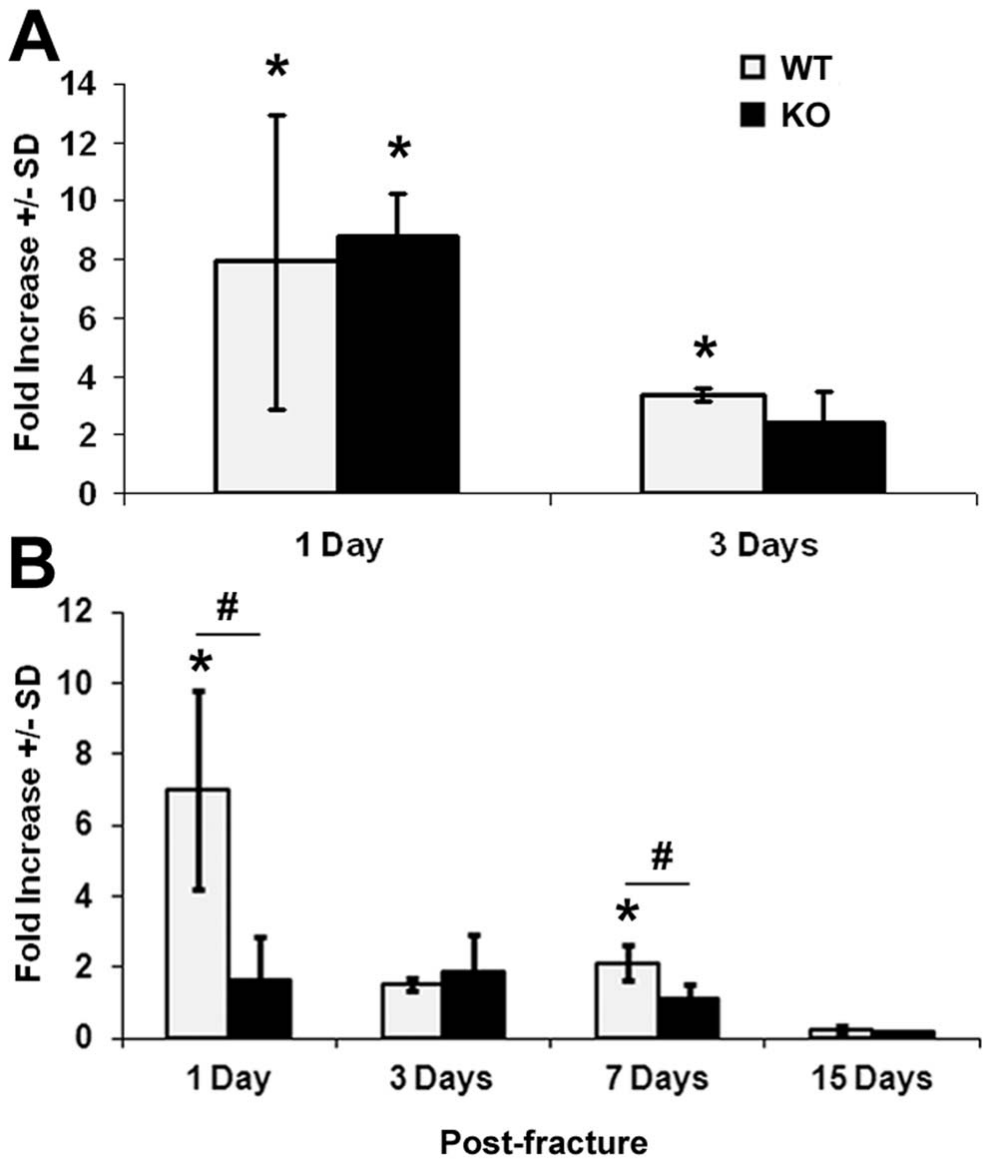
Expression of chemokine genes at four time points post-fracture. **A**. *Ccl3*, **B**. *Ccl2*. Data are expressed as fold-change in the expression of the gene in the fractured bones compared to WT unfractured bones. We examined 3–4 animals/time point/mouse strain at 1 and 3 days and 5–8 mice at 7 and 15 days post-fracture. *p<0.05 vs WT unfractured bone, #*p*<0.05 between fractured bones of the two lines of mice.

The post-fracture inflammatory response is marked by the infiltration of cells that release inflammatory mediators such as cytokines and chemokines. Thus, to determine if the migration of inflammatory cells was affected by the lack of *Darc* expression in KO mice, we evaluated the expression of two CC chemokines, monocyte chemotactic protein 1 (*Mcp-1)*, also called *Ccl2* and macrophage inflammatory protein 1 (*Mip-1α*), *Ccl3*. The expression level of both genes was increased at one day post-fracture, but the magnitude of increase in the expression was reduced by three days post-fracture (**Fig. 7**), when inflammation starts to subside. The expression of *Ccl2* but not *Ccl3* was reduced in *Darc*-KO compared to WT mice at one day post-fracture (**Fig. 7A, B**).

Since *Il-6* and *Ccl2* are the genes that showed the biggest difference in mRNA expression both between fractured and unfractured bones and between the two lines of mice after fracture, we have evaluated the expression of *Il-6* (**Fig. 6**) and *Ccl2* (**Fig. 7B**) at additional post-fracture time points. While the increase in the expression of *Il-6* in response to fracture was greater in WT compared to KO mice at 1 and 3 days post-fracture, no difference was observed at 7 days post-fracture between the two lines of mice when inflammation normally has subsided. Though mRNA expression of *Ccl2* in the fracture calluses decreased at 7 days, it remained significantly greater in fractured bones compared to unfractured bones in WT mice. Furthermore, *Ccl2* expression in fracture calluses derived from WT mice was greater at 1 and 7 days post-fracture compared to fractures derived from KO mice.

**Figure 8:**
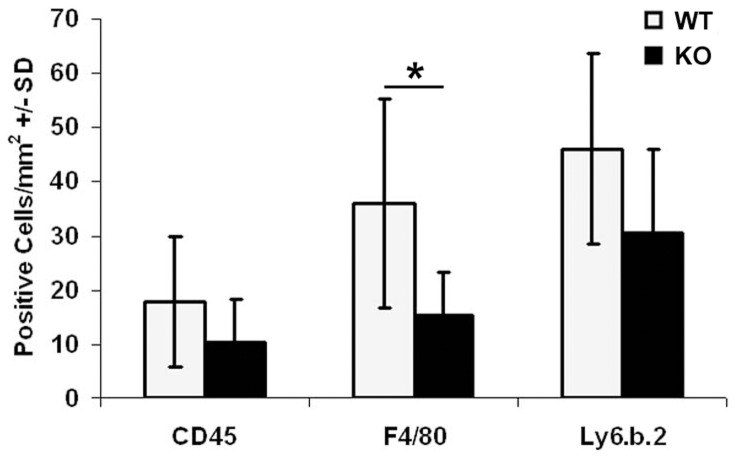
Evaluation of the inflammatory cells around the fractures in *Darc*-KO and WT mice at one day post-fracture. Data are expressed as number of cells/mm^2^ of fracture callus. We examined 5–7 animals/mouse strain. The inflammatory cells were identified by antibodies specific for CD45 (B-lymphocytes), F4/80 (macrophages) and Ly6.b.2 (neutrophils). *p<0.05 WT vs *Darc*-KO mice.

To determine if the expression of cytokines and chemokines is associated with infiltration of inflammatory cells to fractures, we quantified the inflammatory cell population in the bone marrow and soft tissues around the fracture sites (**Fig. 8**). At one day post-fracture, neutrophils were the most abundant and B lymphocytes were the least abundant at the fracture site (data not shown). While the expression levels of markers of neutrophils (Ly-6B.2), B-lymphocytes (CD45R) and macrophages (F4/80) were reduced at the fracture site of *Darc*-KO mice compared to WT mice, the reduction was statistically significant only for the macrophage marker, F4/80 (*p* = 0.04, **Fig. 8**).

## Discussion

Unlike typical chemokine receptors, *Darc* is present on both red blood and endothelial cells but not on leukocytes [30], [31], [32]. Previous studies have shown that *Darc* is crucial for chemokine-mediated leukocyte migration *in vivo*, for the changes in chemokine and chemokine receptor homeostasis associated with *Darc* gene deficiency, and it exerts strong anti-inflammatory effects [33]. Fracture repair is a local event regulated by locally expressed inflammatory mediators. The onset of acute inflammation initiates the early stages of fracture repair and its resolution promotes cartilage formation immediately thereafter, so it would stand to reason that *Darc* regulates this process. However, the involvement of *Darc* in post-fracture inflammation and fracture repair has never been investigated. Therefore, in this study we tested the effect of targeted disruption of *Darc* expression on femoral bone fracture repair and post-fracture inflammation.

We have evaluated local inflammation at one day post-fracture, when the predominant cells in the hematoma are neutrophils [34]. Recruited neutrophils are thought to mediate this switch by producing several macrophage chemoattractants, such as CCL2 and IL-6 [35], [36]. Thus, since lack of *Darc* expression was previously reported to reduce the infiltration of neutrophils to local inflammation site [37], we expected fractures derived from *Darc*-KO mice to show less chemokine and cytokine expression/secretion in fracture tissues, and consequently less macrophage infiltration to the fracture calluses.

*Darc*-KO mice showed a significant increase in cartilage abundance in the fracture calluses at 7 days post-fracture, which was associated with an earlier induction of Collagen-II gene expression and Collagen-X expression in fracture repair in the *Darc*-KO mouse compared to WT strain (**Fig. 3**). These data suggest that fracture callus cartilage has developed earlier in *Darc*-KO than in WT mice, though there is no evidence that *Darc* directly regulates collagen gene expression. It has been reported that inflammation alters chondrocyte proliferation [38] as is observed with persistent inflammation in conditions, such as infection where cartilage development can be impaired. Inflammatory mediators such as IL-1β have a pivotal role in sustaining both inflammation and cartilage erosion, at least in the pathology associated with arthritis [39], [40], [41], [42]. It is known that chemokines can induce cartilage degradation [43], [44], [45], so it is possible that lack of *Darc* expression in the KO mice affected fracture cartilage development through reduced inflammatory cell recruitment and chemokine expression that favored cartilage formation in *Darc*-KO fracture calluses.

The increased cartilage development that occurred very early in fracture repair and well before maximum bone remodeling in *Darc*-KO mice compared to WT mice possibly was associated with reduced post-fracture inflammation in *Darc*-KO fractures. However, the early cartilage formation in *Darc*-KO mice did not translate into accelerated conversion to bone and early bony union, since micro-CT measurements of the hard callus did not reveal any significant differences in the volume of the newly formed bone between the two lines of mice at 21 days post-surgery (**Fig. 4**). Our previous studies demonstrated reduced resorbing surfaces in unfractured bones derived from *Darc*-KO mice compared to WT mice [8], which would be expected to reduce osteoclast recruitment and subsequently delay cartilage resorption in *Darc*-KO fracture calluses compared to WT mice. If cartilage resorption was delayed in *D*arc-KO fractures, its effects on the development of the bony fracture callus were not significant, and we conclude from the cartilage abundance and collagen gene expression differences that the increased fracture cartilage was the result of differences in chondrocyte development. These results suggest that *Darc* deficiency reduces the inflammation that would normally delay cartilage development and Collagen-II gene expression until inflammation is resolved, normally after the first three days post-fracture.

To investigate inflammatory cell infiltration to the fracture tissues, we compared the numbers of inflammatory cells around the fractures in the two lines of mice using antibodies specific for markers of neutrophils, macrophages and B lymphocytes. We quantified the cell numbers at one day post-fracture, because we had observed a tremendous decrease in the expression of the inflammatory cytokines and chemokines in the *Darc*-KO fractures at one day post-fracture (**Fig. 6** **and** **7**). An evaluation of these three cell types revealed a trend toward reduced numbers of these cells in the fracture calluses from *Darc*-KO, although only macrophage numbers were significantly reduced in *Darc*-KO fractures compared to WT fractures (**Fig. 8**). We believe that the reason why we did not reach significance between the two lines of mice for neutrophils was that the size of the groups was insufficient to detect significance with the modest differences in cartilage formation and chondrocyte-specific collagen gene expression between the two lines of mice (Figures 2 and 3, respectively). As expected, neutrophils were the most abundant cells in the fracture tissues at one day post-fracture, as these cells are known to migrate to the injured areas immediately after injury, and as the first inflammatory cells to reach sites of tissue injury, they secrete cytokines and chemokines that play crucial roles in attracting other cells to the injured tissue. They are followed by macrophages that act as phagocytes to clear the wound of matrix and cell debris [46]. Each of these inflammatory cells secrete growth factors and cytokines that are presumed to function as tissue repair signals and direct resident cell functions in fracture callus development during healing. It follows that if the number of neutrophils is reduced in *Darc*-KO fracture calluses; the recruitment of the other inflammatory cells to the fracture will be reduced and will affect healing. Therefore, we speculate that down-regulation of cytokine expression level due to delayed recruitment of inflammatory cells to the fracture callus in *Darc*-KO mice could be responsible for the early cartilage formation in KO mice fractures.

Though the expression of the *Opg* and *Rankl* markers of osteoclast development was significantly greater in fracture calluses compared to unfractured bones in both lines of mice, no difference was observed between the two lines of mice at 7 days post-fracture (**Fig. 5**), suggesting that local osteoclastogenesis was not affected in *Darc*-KO fracture calluses. Since our previous studies have established that bone resorption is reduced in the unfractured bones of the *Darc*-KO mice [8], we propose that cartilage resorption at the fracture site of *Darc*-KO mice could be delayed due to reduced recruitment of osteoclast precursors to the fracture calluses in KO mice compared to WT mice. However, without further studies on osteoclast development and recruitment during fracture healing we cannot conclude that fracture callus remodeling was reduced in *Darc*-KO mice.

Most of the previous studies on *Darc* regulation of inflammation were based upon LPS challenges, chemokine injections or chronic diseases [37], [47], [48], [49]. Dawson et al., [47] reported that there was no difference between the WT and *Darc*-KO mice in LPS-induced leukocyte recruitment to the peritoneal cavity which was the injection site at two hours post-LPS injection, but they noticed more leukocytes accumulation in the lung and liver in the KO mice compared to WT mice. Lee et al., [37] have found that mice that lack *Darc* in erythrocytes showed reduced neutrophils in lung airspaces compared to WT control mice 4 hours after intratracheal instillation of LPS. However, the MIP-2 concentration was found to be higher in the lung airspaces of mice that lack *Darc* expression in both erythrocytes and endothelial cells and from mice that lack *Darc* expression only in endothelial cells but not in erythrocytes at 4 hours (h) but not 2 h post LPS injection compared to control mice. In lung tissue vascular compartment, the concentration of MIP-2 was greater only in mice lacking *Darc* in erythrocytes at 2 h but not 4 h post-LPS injection compared to WT control mice. However, in the plasma, MIP-2 concentration was reduced in *Darc*-KO mice lacking *Darc* in endothelial (4 h) and in erythrocyte cells (2 h) compared to WT control mice. In addition, Fukuma et al., [48] have shown that plasma concentrations of eotaxin (*Ccl11*) and MCP-1 (*Ccl2*), the two chemokines that bind to *Darc*, were significantly lower in *Darc*-KO compared to WT mice in physiological conditions. The clearance from the blood circulation after intravenous injection of these two chemokines was faster in *Darc*-KO compared to WT mice. These data suggest that the lack of *Darc* expression enhances the clearance of chemokines from blood circulation, and that chemokine-*Darc* regulated secretion of soluble chemokines is time- and tissue- dependent, two variables that were different in the healing fracture calluses of our study.

Other studies on fracture repair have established that the acute inflammation that normally initiates fracture repair is a local phenomenon [50] and that a systemic inflammatory response to traumatic injuries impairs fracture healing [51]. We therefore expect the inflammatory response to bone fracture to be local, and that systemic measurements of cytokines in bone repair would be misleading. Our evaluation of post-fracture inflammation was performed at the site of the fracture not in any other organ; we did not evaluate the circulating chemokines or leukocytes after fracture. Because we limited our evaluation to the local fracture tissues and did not examine systemic inflammation, we expect that *Darc* regulation of fracture inflammation would differ from the studies on the systemic effect of *Darc* expression.

Results from human studies suggest that *Darc* expression might function in human fracture repair as it did in the mouse fracture model. It has been demonstrated that rs2814778, a *Darc*-deficient haplotype known to protect the African-American population from malaria infection, was related to low white blood cell and neutrophil counts [52]. However, to our knowledge, only one study compared fracture healing between different ethnicities [53], and they did not find any differences in fracture healing, although they noticed small differences in pain scores during fracture healing. Further investigation with larger patient populations is required to characterize the effect of *Darc* deficiency on fracture healing in human populations.

Based on the previous studies and the present study, we hypothesize that *Darc* delays the clearance of chemokines from the blood circulation and induces the inflammatory cell migration to the injured areas. In the absence of *Darc*, chemokines are rapidly cleared from the blood circulation and are probably retained in the lung and liver which delays the recruitment of inflammatory cells to the injured areas. Taken together, our findings suggest that *Darc* expression promotes the resolution of inflammation in fracture repair. Furthermore, our previous studies have established that bone resorption is reduced in the unfractured bones of the *Darc*-KO mice [8].These data suggest that the delayed cartilage resorption at the fracture site of *Darc*-KO mice could also be due to reduced recruitment of osteoclast precursors to the fracture calluses in KO mice compared to WT mice.

## Conclusions

We have shown for the first time, using mice with targeted disruption of the mouse *Darc* gene that *Darc* plays a role in modulating the regulation of inflammatory response to bone injury and that lack of *Darc* expression promotes cartilage formation in fracture calluses but does not affect bony union or fracture healing at 21 days post-fracture.

## Supporting Information

Table S1 **Information on the primers used for quantitative PCR.**
(DOC)

Table S2 Real Time PCR. Raw Data. Real Time PCR was done using cDNA prepared from RNA isolated from fracture calluses derived from Darc-KO and wild type mice at three time points post-fracture.
(XLS)
