# Supplementary material for: Duffy Antigen Receptor for Chemokines Regulates Post-Fracture Inflammation
Source: PLoS One. 2013 Oct 17;8(10):e77362. doi: 10.1371/journal.pone.0077362 (PMC3798395; doi:10.1371/journal.pone.0077362)
Supplement: Table S1 — Information on the primers used for quantitative PCR. (DOC) [file pone.0077362.s001.doc]

**Supplemental Table S1.** Information on the primers used for quantitative PCR.

| **Primer ID** | **Target gene name** | **Primer Sequence (5’-3’)** | **Seq. covered (bp)** | ACCESSION |
| --- | --- | --- | --- | --- |
| ***Col 2*-F**  ***Col 2*-R** | Collagen, type II, alpha 1 | TGGCTTAGGGCAGAGAGAG  GGTGGCAGAGTTTCAGGTC | 3699- 3899 | NM_001113515 |
| ***Col 10*-F**  ***Col 10*-R** | Collagen, type X, alpha 1 | TTCTGCTGCTAATGTTCTTGACC  GGGATGAAGTATTGTGTCTTGGG | 98-212 | NM_009925 |
| ***Rankl*-F**  ***Rankl*-R** | Receptor activator of nuclear factor-κB ligand | GCAGAAGGAACTGCAACACA  GATGGTGAGGTGTGCAAATG | 532-661 | NM_011613 |
| ***OPG*-F**  ***OPG*-R** | Osteoprotegerin | ACTCGAACCTCACCACAGAGCA  GTGCTTGAGGGCATACATCAGG | 1097-1291 | NM_008764 |
| ***Tnfa*-F**  ***Tnfa*-R** | Tumor necrosis factor alpha | CCCTCACACTCAGATCATCTT  GCTACGACGTGGGCTACAG | 386-448 | NM_013693 |
| ***Il-1b*-F**  ***Il-1b*-R** | Interleukin 1 beta | CTTCAGGCAGGCAGTATCACTC  TGCAGTTGTCTAATGGGAACGT | 245-439 | NM_008361 |
| ***Il6*-F**  ***Il6*-R** | Interleukin 6 | CATCCAGTTGCCTTCTTGGG  CCAGTTTGGTAGCATCCATC | 59-339 | NM_031168 |
| ***Mip-1*-F**  ***Mip-1*-R** | Macrophage inflammatory protein 1 alpha, also called Ccl3 | CTCCCAGCCAGGTGTCATTTT  TTCCTCGCTGCCTCCAAGACT | 272-402 | NM_011337 |
| ***Mcp-1*-F**  ***Mcp-1*-R** | Monocyte chemotactic protein 1, also called Ccl2 | TTAAAAACCTGGATCGGAACCAA  GCATTAGCTTCAGATTTACGGGT | 345-465 | NM_011333 |
